# Community health volunteers challenges and preferred income generating activities for sustainability: a qualitative case study of rural Kilifi, Kenya

**DOI:** 10.1186/s12913-021-06693-w

**Published:** 2021-07-03

**Authors:** Adelaide M Lusambili, Njeri Nyanja, Sophie Vusha Chabeda, Marleen Temmerman, Lucy Nyaga, Jerim Obure, Anthony Ngugi

**Affiliations:** 1 Department of Population Health (DPH), Aga Khan University, Nairobi, Kenya; 2 Family Medicine, Aga Khan University, Nairobi, Kenya; 3 Centre of Excellence in Women and Child Health, Aga Khan University, Nairobi, Kenya

**Keywords:** CHV, Attrition, Challenges, Sustainability, Kenya

## Abstract

**Background:**

There is a global emphasis on engaging community health volunteers (CHVs) in low- to middle-income countries (LMICs) to reach to the vast underserved populations that live in rural areas. Retention of CHVs in most countries has however been difficult and turnover in many settings has been reported to be high with profound negative effects on continuity of community health services. In rural Kenya, high attrition among CHVs remains a concern. Understanding challenges faced by CHVs in rural settings and how to reduce attrition rates with sustainable income-generating activities (IGAs) is key to informing the implementation of contextual measures that can minimise high turnover. This paper presents findings on the challenges of volunteerism in community health and the preferred IGAs in rural Kilifi county, Kenya.

**Methods:**

The study employed qualitative methods. We conducted 8 key informant interviews (KIIs) with a variety of stakeholders and 10 focus group discussions (FGDs) with CHVs. NVIVO software was used to organise and analyse our data thematically.

**Results:**

Community Health Volunteers work is not remunerated and it conflicts with their economic activities, child care and other community expectations. In addition, lack of supervision, work plans and relevant training is a barrier to delivering CHVs’ work to the communities. There is a need to remunerate CHVs work as well as provide support in the form of basic training and capital on entrepreneurship to implement the identified income generating activities such as farming and events management.

**Conclusions:**

Strategies to support the livelihoods of CHVs through context relevant income generating activities should be identified and co-developed by the ministry of health and other stakeholders in consultation with the CHVs.

**Supplementary Information:**

The online version contains supplementary material available at 10.1186/s12913-021-06693-w.

## Background

The use of Community Health Volunteers (CHVs) to reach underserved communities has gained increased recognition in the past two decade [1–3]. CHVs are an important nexus between the community and the formal health system. Globally, a plethora of literature have reported a link between CHVs and health improvement [1–7]. Studies from sub-Saharan Africa illustrate that, where CHVs are actively engaged, maternal and neonatal mortality are reduced and positive health behaviours and access to maternal new child health (MNCH) care are enhanced [1, 3, 4]. Other studies have shown that, CHVs play a pivotal role by providing linkages between hard to reach communities and facilities, educating pregnant women of the importance of delivering at the facility, and increasing community referrals [8, 9]. Distinctively, research has alluded that, attrition rates remain high in sub-Saharan Africa where despite greater needs, CHVs face an increased workload characterised by lack of financial remuneration, poor infrastructure, lack of supervision and high levels of isolation [10–14].

Kenya, like many other low-income countries struggles with a fragile health system [15]. Many rural areas, where 70% of the population live, remain disconnected from mainstream health services. Despite mounting evidence of the positive results realised by CHVs, attrition is high among these volunteers.

Community Health Volunteers are chosen by Community Health Committees (CHCs) and Community Healthcare Assistants [CHAs]. According to the Kenya Community Health Policy 2020–2030 [16], CHVs are supposed to be selected on the premise that they reside within the communities in which they serve, they can speak the local languages, they have acquired primary education, can read and write and understands that their role is an unremunerated. Community Health Assistants are charged with the responsibilities of supervising, providing technical support as well as training them using an accredited training curriculum. It is CHAs duty to ensure that CHVs have identity and visibility labels during their work in the communities.

Aga Khan University–Nairobi has been conducting health research and programming work in the Kilifi county in coastal Kenya. Through this work we have noted that, CHVs play a fundamental role in promoting the health of the vast majority of the marginalized populations that live in the rural Kilifi county. Often, however, continuity of services cannot be maintained as the turnover among CHVs is high [17]. Kenya’s budget for health care personnel has historically been minimal, and thus not adequate to support CHVs adequate provision of healthcare services. In geographically dispersed communities  like Kilifi, CHVs may need additional support with transportation cost –in rare cases, this has been addressed through Community Based Organisation (CBOs) partners or development partner funding which has proven to be an unsustainable strategy. This complexity calls for renewed thinking about mechanisms for promoting context-based, informed  and , sustainable interventions within the local health systems. Strengthening CHVs work in rural Kenya is dependent on motivation which is determined by incentives. In Kenya, this is poorly studied. We conducted this study to examine the socio economic challenges that affect the volunteer work of CHVs and to explore potential incentivisation strategies through group based income generating activities.

## Methods

### Study design

This study adopted an exploratory qualitative approach. We conducted focus group discussions with CHVs and key informant interviews (KIIs) with key stakeholders, from the Ministry of health, Ministry of Agriculture (MOA) and multilateral IGA stakeholders representing the two sub-counties of Kilifi.

### Study setting

The study was conducted in Kaloleni and Rabai sub-counties in Kilifi in the Coast of Kenya. The study area has an on-going health programming relationship with the Faculty of Health Sciences of Aga Khan University–East Africa. At the time that the data were collected, Aga Khan University was conducting a maternal, newborn and child health (AQCESS^1^) project which was funded by the Canadian government through Global Affairs Canada (GAC) with co-funding from the Aga Khan Foundation Canada (AKFC). The AQCESS project aimed to accelerate the reduction of maternal and child mortality. While the study was nested in AQCESS project which purely focused on RMNCH, the CHVs interviewed had nothing to do with the ongoing AQCESS activities.

Kaloleni and Rabai are among the poorest Sub-counties in Kenya, relying on subsistence agriculture and tourism. The temperatures are high throughout the year, with daily temperatures averaging above 23 °C, adding to the burden of food scarcity as the local people have few options for crops that can thrive in this environment. The health outcomes of the population are generally poor, in part due to poverty, illiteracy, limited health infrastructure. Women utilise the services of traditional birth attendants (TBAs) during delivery and pregnant women seeking care at facilities do so only for the actual delivery [18]. Importantly, in Kilifi, villages are far apart from one another, implying that distances from medical facilities is far. This necessitates an urgent need to train, empower and sustain the CHVs to serve the isolated communities, women and children who have limited access to facility-based care.

### Study population and sampling

We conducted 10 focus group discussions with CHVs and eight interviews with key informants. To obtain a grounded understanding of the challenges and possible enablers to CHVs’ work in the two sub-counties, the study team considered CHVs an appropriate group for study as they would provide their lived experiences working in the communities and their views on what would work to empower them to continue their work. The CHVs participating in the FGDs were selected within Rabai and Kaloleni sub-counties and were designed to represent 10 Community Health Units (CHUs).^2^ In total we had seven FGDs from Kaloleni and three FGDs from Rabai with equal gender distribution within each CHU. Table 1 below summarises the characteristics of the CHVs participating in the study.

**Table 1:** Demographics for the focus group discussions (FGDs)

1	GENDER	• Female CHVs interviewed were 64 • Males CHVs interviewed were 17
2	EXPERIENCE	• All CHVs had worked for more than one year.
3	AGE	• CHVs interviewed ranged from 26 to 67 years old
4	EDUCATION LEVELS	• All CHVs interviewed had attained primary level of education and could write and read
5	RESIDENCY	• We interviewed 81 participants of which 57 were from Kaloleni and 23 were from Rabai sub-counties

Key informant interviews included one County Ministry of Health (MOH) official, four sub-county MOH officials, two Ministry of Agriculture (MOA) officials and two multi-lateral stakeholder representatives from Kaloleni and Rabai sub-counties. Key informant categories were sampled by the study team with the help of local leaders and all participants had over 2 years of experience in their roles. Table 2 below provides a summary of all the KIIs interviewed for the study and a justification for their participation.

**Table 2:** Demographics for the KIIs

Participant	Justification
Ministry of health sub-county officials *N* = 2	We interviewed Kaloleni and Rabai sub-county ministry of health officials to provide views on policies and future plans on CHVs sustainability. We also wanted to understand what they know and what strategies and measures are in place to enhance the sustainability of the work of CHVs
Ministry of Agriculture-Sub County *N* = 2	Considering that the people in Kilifi area are subsistence farmers, we interviewed agricultural extension officer from Rabai and Kaloleni with specific experience in community extension to provide insights on agriculture-based IGAs appropriate for the Kilifi ecology.
Income Generating Activity Trainer (IGA] – CBO *N* = 2	We interviewed IGA from both Kaloleni and Rabai who were conversant with training community based organisation on entrepreneurial activities funded by the government. We hoped that they would share their experience on the potential opportunities and challenges.
Stakeholder/CBO	The chairperson of a local CBO group –Upendo in Rabai was interviewed due to his distinct role in local CBOs as well as promoting IGAs.
Stakeholder/CBO	A CBO youth leader in Rabai was interviewed based on his experience working with the youth on IGAs.

### Data collection

Data collection commenced after ethical approval was obtained from the AKU Institutional Ethics Research Committee (AKU–IERC) and the National Commission for Science, Technology, and Innovation (NACOSTI). We also obtained permission from the Kilifi county office and from local leaders in Kaloleni and Rabai sub-counties.

The study guides for the FGDs and KIIs were developed in English by the principle investigator (PI).

These guides were reviewed by a team of research scientist –and then translated to Kiswahili. Two research assistants underwent   a two  days training to familiarise themselves with the study objectives and the study guides. The study guide was piloted in Kaloleni in two FGDs with 16 CHVs who were not part of this study. Concerns raised after piloting were discussed by the research team, and items were reworded with some added and others omitted as necessary.

All participants were given information about the study. The study team explained issues of confidentially, voluntary participation, risks involved and benefits for the study. Information sheets and informed consent followed the international and local principles based on the Declaration of Helsinki. All FGDs and KIIs interviews began after receiving signed informed consent forms from the participants.

Data collection was led by an experienced qualitative research facilitator (SC). All FGDs were conducted in Kiswahili and English language as preferred by the  participants, in a convenient health facility and community centres, private enough to allow conversations. The FGDs were mixed gender and were all audio recorded. Data collected included socio demographic information, challenges encountered, potential engagement in income generating activities, preferred income generating activities and proposed sponsors. All FGDs lasted between 40 to 100 min, followed by a debrief session from the facilitator.

Key informant interviews were conducted at a convenient time and place within the offices in which the participants worked. They were conducted in English, and lasted for 40 to 60 min. Data captured from the KIIs interviews were participants perceived challenges that contributed to CHVs high attrition, their views about financial contribution, existing policies, access to support and their insights  on the need to remunerate CHVS.

### Data management and analysis

The audio recording was backed up securely and encrypted by the Monitoring and Evaluation Research Learning (MERL) unit at Aga Khan University. Data from the audio tapes were transcribed verbatim, with names and identifiers removed, and translated back into English language for analysis. Both inductive and deductive data analysis were conducted. First, the principal investigator (NN) reviewed all the transcripts, became familiar with the data and generated the initial codes. Second, for rigor and validity, two researchers (NN, SC) reviewed the data and further developed the initial codes through indexing and charting and reached consensus. Third, an independent researcher (AL) reviewed the transcripts, coded the responses and identified themes. Finally, SC, NN, AL and AN compared the coding and emerging themes, resolved discrepancies and agreed on the themes presented in Table 1 below. A consolidated criterion for reporting qualitative studies (COREQ) was followed [19].

#### Findings

As illustrated in Table 3 below, part A of this study presents findings on the challenges faced by CHVs. Part B presents the income-generating activities interviewees wished to undertake that could help to enhance sustainability of involvement in community health work as CHVs. Findings reported in this paper are largely from the CHVs, as data from the key informant interviews did not make meaningful contributions to CHVs lived experience with regard to challenges faced and preferred income generating activities. However, data from KIIs provided data on policy, and that will be reported in a separate paper.

**Table 3:** Codes, categories and themes

	Codes	Categories	Themes
A	-We are volunteers -CHV work conflicts with my informal business -CHV work conflicts with childcare; I can’t pay for childcare -I close my business to do CHV work -I can’t do CHV work with full-time job -It is difficult to attend social functions; -CHV work conflicts with household chores	-Lack of incentives -Conflicting chores -Lack of adequate time to do CHV work -Planning and time management	Effectiveness of CHVs’ work hampered by social-economic factors Context-specific challenges
B	-We have no work plans -Unplanned meetings -CHV have no certification	-Poor coordination -Poor planning -Lack of training	Lack of training and daily work plans
C	-People don’t understand who we are -People don’t understand what we do -We need labelled badges; we need labelled T-shirts -we have nothing to show that we are CHVs	-Need for identity -Pride of being a CHV -Recognition -A sense of belonging -Lack of identity and insecurity; identity can lead to trust	Identity/identification and trust
D	-We prefer to buy chairs, tents and cutlery to use during weddings and funerals -high demand for chairs during weddings -high demand for chairs during funerals; chairs and tents needed for weddings and funerals -we have many weddings -hiring chairs and tents is a sustainable IGA; -chicken-rearing; kitchen gardens	-Events management; contextually supported activities (hiring of chairs and tents) -Farming, poultry-keeping	Income generating activities more likely to enhance retention among CHVs in Kaloleni and Rabai sub-counties -Hiring of tents, chairs and cutlery widely preferred and supported by contextual factors, e.g., high demand during weddings and funerals • Some places suitable for chicken-rearing

#### Part A: challenges faced by CHVs in Rabai and Kaloleni

##### Competing economic and social-cultural demands

This study found that economic and social-cultural demands interplay with competing priorities within the same context of delivering CHVs work. Across the FGDs in both Kaloleni and Rabai, CHVs noted that they were not paid and were doing health promotion work in the communities alongside other chores, such as child care, managing their small business and attending to other community functions, making CHV work challenging.

CHVs noted that they are often required to be in two different places at the same time

*The challenge that I experience sometimes is because I need to be in a seminar and at the same time it is time to farm and remove weeds from maize. Now you find that you come here [doing CHV work] a whole week and you miss on the other side[Farming]* ***FGD 1–RABAI***

*I am someone who sells Lesos [local clothes business]. There could be a date that is set for us to hand in the report [community health report] and maybe there is an activity that has come up that involves death [participation in community funerals]… Now you have to plan yourself—do I take the report [community health report] or go for the sale activity[local clothes business]? So, now if you take the report, there will be no business for you on the other side, I will have missed my customers.* ***FGD 5–KALOLENI***

In rural Kaloleni and Rabai, most people run small informal businesses that are competitive and requires them to open their business premises throughout the day. In addition, employing someone to manage these businesses is expensive. CHVs with small informal businesses find it challenging to balance operating their businesses daily with community health promotion work.

*I am required to be at my business and at the same time I need to be offering community service in connection with these health issues. Often, I have to look for someone to manage my business so [that] I can continue with these health care activities****. FGD 1–RABAI***

*You are supposed to move around, maybe you have opened your business [and] you have to close it. … When you close it, the buyers come, they find you closed it, they go away.* ***FGD 5–KALOLENI***

Child care needs competed with CHVs work. CHVs faced an additional burden to pay for child care in the midst of performing an unremunerated job.

*For now, I have a grandchild whom I am taking care of. My challenge is, I have to look for someone [to] leave my grandchild with before I can go to the community. Sometimes I leave her [childcare] and tell her, ‘I will give you anything that I get [payment]. Or sometimes when I am called for a seminar and I leave the same person [child care] with my grandchild, she also hopes to get something [to be paid].* ***FGD 4–RABAI***

The arrangement, coordination and planning of CHVs work make it difficult for members to take formal work. CHVs who managed to get casual jobs found it difficult to continue with their work

*So, the challenge I get from my individual work and the CHV is that, maybe I leave here and go to Mariakani (town), I get a contractor [employer] …, he contracts me for 3 weeks, that means no CHV work as I will be there with the contractor who tells me to finish this work....and here [at the same time] the reporting time [for community health report] has reached. ..this is a challenge.* ***FGD 9–KALOLENI***

##### Structural challenges

The interplay between lack of training, poor coordination and daily planning have a direct bearing on CHVs work. CHVs are hired within the communities in which they live through recommendation by community public health officers at the village levels. The training undertaken by CHVs is not clear as many of them may assume roles they have not been trained for. For instance, CHVs roles entail promoting reproductive maternal and newborn health (RMNCH), community referrals and counselling. To sufficiently perform these roles, training on varied aspects of their roles and clear daily and weekly work plans is required. Observations from a key informant indicates a lack of training and work plans. CHVs may not have the necessary training nor proper work plans.

*Okay I think it will be best if these CHVs can undergo thorough training first to enhance whatever they have, the information they have. Secondly, they could make proper work plans which could be financed to do the outreaches.* ***KII–Community Based Youth Leader***

Further observations from a key informant for the study observed that the assigned number of CHVs in each community may be small, making it difficult to plan for shifts. These is compounded by lack of certification of their role and economic challenges.

*In order for them to be effective, they must be properly facilitated other than that their number in a given centre must at least be a good number. Then you find in those health centres maybe they have one or two CHVs going on a shift.* ***KII, Extension officer***

*So, I understand that they are trained but the only problem that those people [CHVs] are lacking is the certification.* ***KII, Ministry of Agricultural representative***

*There is the economic challenge … now they keep questioning about what do they get in return after that, after doing that … since it is something voluntary. KII –Community Leader*

Findings from Kaloleni and Rabai reveal that CHVs’ meetings are sometimes unplanned. Inadvertent meetings may interfere with individuals’ overall daily plans, including their businesses and how they feed their families.

*The challenges that I am experiencing, for example, I do small business, I have made my Mahamri [local cakes] in the morning [to sell] and then I find out there is an impromptu meeting for the CHVs. I have to leave my things [business].* ***FGD 2–RABAI***

*Like today, I was to plant my tomatoes, I was to start at 2 o’clock, to dig the holes, put the manure at 5 o’clock in the holes, but that was impossible. I left it and came here at 12 o’clock until now I am here* ***FGD 6–KALOLENI***

*My potato frying business [is] in the evening or making porridge in the morning. … If I am needed somewhere, my business has to close. …those potatoes usually get spoilt, they won’t find another person to sell them.* ***FGD 6–KALOLENI***

##### Identification and trust

The data suggest that in some instances CHVs may not be recognized in the communities, in part because they do not have the right training and uniforms. The data also suggest that CHVs felt that identification would give them a sense of pride and respect in the communities in which they worked.

*If we as community health volunteers, we can be recognized as providing health in the community, we can get a badge, t-shirt so we get in the community they know these are the health volunteers, because there are places when we get there we are despised, because we go just with our clothes as usual and then if you tell them to dig a toilet [pit latrine] they tell you go tell the doctor who sent you to come and dig that toilet [pit latrine] for them. But if we have the approvals[a badge and uniforms], …they will give us the respect and even our work will continue on well.* ***FGD 2–RABAI***

*We would like to have uniform...we have our uniforms, we have our bags…then people will respect us, they will say these women are working at the hospital.* ***FGD 6–KALOLENI***

#### Part B: possible income-generating activities

Part B presents information from study participants about preferred IGA activities that could be implemented to improve their economic well-being and lead to increased sustainability. Events management and farming were considered most preferred forms of IGA in this context.

##### Events management as an IGA

Narratives from the CHVs across all the FGDs suggest the high demand to supply equipment, furniture and other necessities required during social-cultural functions such as weddings, funerals and birthdays. Participants felt that they could fill this gap if they were given financial assistance to purchase and stock most of these items and rent them out during functions.

*If we were given an opportunity to choose the projects that we would like to develop later, we would like to get tents, cooking pots, plates, the cups considering that there are seasons where activities become more like weddings, funerals and stuff like these are widely used, so I feel we can earn money for the development of our [CHV] group ….* ***FGD 1–RABAI***

*I feel the chair project together with the tents and the food trays, it [is] a project that can lift us up quickly because there are many activities and those things are hired out and are used let’s say in every week they are hired out and used, especially these tents and plates and these chairs. So, I feel that one can lift us up quickly.* ***FGD 3–KALOLENI***

##### Farming and poultry keeping

Agricultural IGAs were considered relevant among the participants. Data suggests that participants could benefit from financial help either to buy communal land for farming or start chicken rearing projects.

*I would also like to have land that we could till as a community unit and then just be helped with the seeds and fertilizers, so we can strengthen the farming and we can benefit from it.* ***FGD 2–RABAI***

*For our case, maybe let us think of a crop like cassava. Cassava is a crop, isn’t it? It has got various chains from the cuttings maybe you want to make products, make some crisps, maybe you want to make cassava flour, and so forth.* ***KII–Extension officer***

Participants talked of the need to get a financial sponsor to help them in achieving their aspirations of starting farming projects.

*I would want chicken-rearing project, the ones that can produce eggs. I would like to have small business like rearing chickens for their meat and cows for milk. …but as I said if I get a sponsor to add for me so that they are many and also, if its food is available, that can be important to me.* ***FGD 3–KALOLENI***

*The chicken-rearing is of two kinds or three kinds. There is farming for organic chicken for meat and eggs. So, I would like to say if we get the sponsor, we get the [chicken] rearing for meat, which is four weeks [grows in 4 weeks], and you sell them, and we also get [chicken] for the eggs. Then later we rear the organic ones because the organic ones also have good market.* ***FGD 5–KALOLENI***

In this section we have shown that community health volunteerism in rural Kilifi is not sustainable due to the interplay between individual and systemic influences. To minimise the rate of attrition, data shows CHVs desire to build on the already existing local activities such as farming and events management to generate income.

## Discussion

Using qualitative approaches, this paper aimed to present the context-based challenges faced by CHVs in rural Kilifi and their preferred IGAs. Our study results revealed that CHVs’ work in Kilifi is an unremunerated, and that it conflicts with CHVs’ other chores at the family and community levels and potential paid employment. These findings correlate with previous research findings in some parts of Kenya and other parts of sub-Saharan Africa, where lack of financial compensation and non-financial material incentives have been reported to be an impediment to the delivery and sustainability of CHVs work [10–14, 16, 18]. The Kenya Community Health Strategy 2020–2030 emphasizes the need for the CHVs to take their roles with a clear understanding that they are voluntary and they must be self - supported. This strategy which is relatively new, pays little attention to variations in Kenya’s rural context where some communities are knit together while other communities like rural Kilifi are geographically dispersed, requiring CHVs to walk over an hour from one community to the other. Kilifi county faces an additional challenge, for instance, temperatures are high above 23 °C, with periods of extended heat stress which may make it difficult for CHVs to deliver their work. It is vital that the county offices should develop innovative measures to motivate the CHVs to conduct their work.

In order for the CHVs run grass-roots health promotion and education programs to reach their mature potential and contribute to the attainment of local and national health goals, it is imperative that the problem of attrition of trained CHVs be addressed using livelihood support interventions that motivate performance and enhance retention. This could be done through financial support to improve their preferred IGAs, which should run independent of their work in the communities.

Moreover, interventions to promote income-generating activities identified in this work should consider Kilifi’s contextuall factors. For example, interviewees see farming, poultry-raising and event management as the key IGAs to be implemented. As the process for this implementation was not discussed, there is need for further participatory research to discuss these approaches to consider viable solutions. Equally important, respondent’s views on IGAs preferences may have been influenced by what they see within their surroundings. It seems clear that CHVs in Kilifi could benefit from support in the form of capital as well as basic training on entrepreneurship to implement the identified IGAs. Implementation models could borrow from other programs that have provided CHVs with preferential access to micro-loans or grants as seed capital for income generating enterprises [18]. When properly implemented, these enterprises will foster entrepreneurship and self-reliance by the CHVs, allaying their anxieties about inability to provide for their families and subsequently increasing motivation. The group-ownership is likely to result in better cohesion and ensure that operational tasks are distributed among the members, thereby reducing competition for time between their volunteer community tasks and responsibilities to their households. Furthermore, Kilifi County departments should collaborate with other departments to implement the identified IGAs as this will create synergy in health promotion with other ministries such as the youth. Co-designing of such interventions with the CHVs and other stakeholders would enhance relevance, acceptability and sustainability.

Findings from this study demonstrates lack of training and poor planning overall for CHVs work. These findings are also reported in other studies [8, 9, 11]. Furthermore, our findings point to the need for CHVs to have clear work plans and work identity by providing them with uniforms and ID badges as a way to build trust, acceptance and recognition in the communities. These findings correspond with conclusions  from other local studies [16]. These   results  exemplify the need for measures to be put in place to routinely develop CHVs capacity through trainings and continuous education to empower them with the knowledge appropriate to the scope of their work. To maintain their motivation and interest in this work, supervision of CHVs by the Ministry of Health should be mandatory, as research shows that many CHVs may work in isolation, with lack of logistical and infrastructural support. The Kilifi Department of Health should address this concern, considering that CHVs work in remote areas and represent an important health systems resource in extending services to undeserved poor communities.

## Conclusions

Community Health Volunteers may find it difficult to strike a balance between their work, the need for economic survival, child care and community functions. Strategies to support the livelihoods of CHVs through context relevant income generating activities should be identified and co-developed by the ministry of health and other stakeholders in consultation with the CHVs. This study, which fostered collaborative identification of preferred and context relevant livelihood support activities is a first step towards such an initiative and should be carried forward to implementation. These findings have increased our knowledge of the challenges faced by CHVs as well and provided insights into possible mechanisms for sustaining their work through IGAs.

### Study limitations and strength

This study is limited to Kilifi context and findings should inform the development of a survey to collect views of CHVs across the country. The study strength lies in the ability to generalise the findings to other counties in the coastal areas of Kenya and in other rural settings within the region. The study findings will also lead to the development of interventions that are contextually informed by the local CHVs and stakeholders. Future research should examine context factors that may motivate CHVs to undertake IGAs involving supply of chairs and table during funerals and weddings.

## Supplementary Information

**Additional file 1.**

## Data Availability

Data cannot be shared publicly as it has participants’ confidential information which may compromise their privacy. Researchers who meet the criteria for access to confidential data can contact the following individuals at the Aga Khan University: Anthony.ngugi@aku.edu; njeri.nyanja@aku.edu and adelaide.lusambili@aku.edu) as well as research.supportea@aku.edu.

## Abbreviations

ANC: Antenatal care
AKU: Aga Khan University
AQCESS: Access to Quality Care through Extending and Strengthening Health Systems
CHV: Community health volunteer
GAC: Global Affairs Canada
FGD: Focus group discussion
IGA: Income-generating activity
KII: Key informant interviews
LMIC: Low- to Middle-Income Countries
MNCH: Maternal new child health
MOA: Ministry of Agriculture
MOH: Ministry of Health
NACOSTI: National Commission for Science, Technology, and Innovation

## Footnotes

1 Access to Quality Care through Extending and Strengthening Health Systems.

2 Under the Kenya government’s Community Health Strategy, the Community Health Unit (CHU) is the lowest level of service delivery, which comprises on average 1000 households (approximately 5000 to 10,000 local population), usually within an administratively and geographically defined area and linked to a level 2 (dispensary) or 3 (health centre) healthcare facility.

## References

[1] Tseng YH, Griffiths F, de Kadt J, Nxumalo N, Rwafa T, Malatji H, Goudge J (2019). Integrating community health workers into the formal health system to improve performance: a qualitative study on the role of on-site supervision in the south African programme. BMJ Open.

[2] Perry HB, Zulliger R, Rogers MM (2014). Community health workers in low-, middle-, and high-income countries: an overview of their history, recent evolution, and current effectiveness. Annu Rev Public Health.

[3] Barnett ML, Gonzalez A, Miranda J, Chavira DA, Lau AS (2018). Mobilizing community health workers to address mental health disparities for underserved populations: a systematic review. Admin Pol Ment Health.

[4] Datiko DG, Yassin MA, Theobald SJ, Blok L, Suvanand S, Creswell J, Cuevas LE (2017). Health extension workers improve tuberculosis case finding and treatment outcome in Ethiopia: a large-scale implementation study. BMJ Glob Health.

[5] Glenton C, Scheel IB, Lewin S, Swingler GH (2011). Can lay health workers increase the uptake of childhood immunisation? Systematic review and typology. Tropical Med Int Health.

[6] Haver J, Brieger W, Zoungrana J, Ansari N, Kagoma J (2015). Experiences engaging community health workers to provide maternal and newborn health services: implementation of four programs. Int J Gynaecol Obstet.

[7] Sepulveda J, Bustreo F, Tapia R (2006). Improvement of child survival in Mexico: the diagonal approach. Lancet..

[8] Kok MC, Broerse JEW, Theobald S, Ormel H, Dieleman M, Taegtmeyer M (2017). Performance of community health workers: situating their intermediary position within complex adaptive health systems. Hum Resour Health.

[9] Kok MC, Kane SS, Tulloch O, Ormel H, Theobald S, Dieleman M, Taegtmeyer M, Broerse JEW, de Koning KAM (2015). How does context influence performance of community health workers in low- and middle-income countries? Evidence from the literature. Health Res Policy Syst.

[10] Aseyo RE, Mumma J, Scott K, Nelima D, Davis E, Baker KK, Cumming O, Dreibelbis R (2018). Realities and experiences of community health volunteers as agents for behaviour change: evidence from an informal urban settlement in Kisumu, Kenya. Hum Resour Health.

[11] Brunie A, Wamala-Mucheri P, Otterness C, Akol A, Chen M, Bufumbo L, Weaver M (2014). Keeping community health workers in Uganda motivated: key challenges, facilitators, and preferred program inputs. Glob Health Sci Pract.

[12] Dil Y, Strachan D, Cairncross S, Korkor AS, Hill Z (2012). Motivations and challenges of community-based surveillance volunteers in the northern region of Ghana. J Community Health.

[13] Glenton C, Colvin CJ, Carlsen B (2013). Barriers and facilitators to the implementation of lay health worker programmes to improve access to maternal and child health: qualitative evidence synthesis. Cochrane Database Syst Rev.

[14] Ormel H, Kok M, Kane S, Ahmed R, Chikaphupha K, Rashid SF, Gemechu D, Otiso L, Sidat M, Theobald S, Taegtmeyer M, de Koning K (2019). Salaried and voluntary community health workers: exploring how incentives and expectation gaps influence motivation. Hum Resour Health.

[15] Chuma J, Okungu V (2011). Viewing the Kenyan health system through an equity lens: implications for universal coverage. Int J Equity Health.

[16] Kenya Community Health Policy 2020. https://www.health.go.ke/wp-content/uploads/2020/07/Kenya-Community-Health-Policy-Signed.pdf. Retrieved 29 May 2021.

[17] Ngugi AK, Nyaga LW, Lakhani A, Agoi F, Hanselman M, Lugogo G, Mehta KM (2018). Prevalence, incidence and predictors of volunteer community health worker attrition in Kwale County, Kenya. BMJ Glob Health.

[18] Lusambili AM, Naanyu V, Wade TJ, Mossman L, Mantel M, Pell R, Ngetich A, Mulama K, Nyaga L, Obure J, Temmerman M (2020). Deliver on your own: disrespectful maternity care in rural Kenya. PLoS One.

[19] Tong A, Sainsbury P, Craig J (2007). Consolidated criteria for reporting qualitative research (COREQ): a 32-item checklist for interviews and focus groups. Int J Qual Health Care.

