# Supplementary material for: Community health volunteers challenges and preferred income generating activities for sustainability: a qualitative case study of rural Kilifi, Kenya
Source: BMC Health Serv Res. 2021 Jul 3;21:642. doi: 10.1186/s12913-021-06693-w (PMC8254366; doi:10.1186/s12913-021-06693-w)
Supplement: Supplementary file 1 — Additional file 1. [file 12913_2021_6693_MOESM1_ESM.docx]

APPENDICES

**Appendix 01: Study information and informed consent form**

**AQCESS PROJECT**

**Appendix 1: Information sheet**

**UNDERSTANDING COMMUNITY HEALTH VOLUNTEERS’ PREFERENCES FOR SOCIO-ECONOMIC EMPOWERMENT STRATEGIES: A QUALITATIVE STUDY IN KILIFI, KENYA**

**Introduction**

Hello, my name is __________________________________and I am working with the AQCESS project of the Aga Khan University, East Africa. Within this project, we are conducting a study among Community Health Volunteers (CHVs) to determine how they would want to be supported to ensure that they remain active in community health work so as to continue promoting the health of their community. Since you are one of the CHVs/CHC member involved in this project/MOH partner in this project/Official of Ministry of Agriculture (MOA) serving in this area/Official of an NGO working in this area, community resource person in this area, *[delete as appropriate]*, I am going to give you information and request you to be part of the study. We would very much appreciate your participation.

**Purpose**

The purpose of the study is to determine what motivational factors would encourage CHVs to remain active in community health service. Specifically, we are interested in finding out if establishing income generation activities for CHVs would motivate them to stay/remain active and what IGAs they would prefer. The information gathered will inform the AQCESS project managers and the government partners.in planning the implementation of IGAs for CHVs in this area

**Participant selection *[delete as appropriate]***

**For CHVs:** You were selected to participate in this study because you are a CHV selected and trained within the AQCESS project.

**Procedures**

The interview/discussion will take between 40 and 60 minutes, will include your demographic information, and questions that address the objective of this study.

**Risks and discomforts**

The study poses no risks to you. However, if a question or issue of discussion causes any anxiety or discomfort you may choose not to answer without giving a reason. If at some point you wish to stop the interview and not to be part of the study please let the interviewer know. Your wish will be granted and will not affect your role as CHV or your relationship with the AQCESS project in any way. You are free not to explain why you would wish to withdraw from the study at that point. However, we hope you will participate fully in the study.

**Benefits**

There are no immediate and direct personal benefits for participating in this study. Your responses in particular and the findings of this study in general will however be incorporated in to the development of AQCESS project IGAs for CHVs, which will be implemented to ensure sustainability of community health programs in your Sub-county.

**Confidentiality**

All information collected during this study will be strictly confidential. The completed questionnaire and signed Consent Form will be kept together. All research materials will be assigned a confidential number for coding purposes. Results will not be released or reported in any way that might allow for identification of individual participants. All information will be aggregated and will be used only for community reference. Your name will not be associated with any of the findings.

**Contact information**

For further information about the study, please contact The Study Principal Investigator (PI) Dr. Njeri Nyanja (0711-092895) or Ms. Lucy Nyaga (0722-208657) or Dr. Anthony Ngugi (020-3662958).

**Consent Statement**

I have read the preceding information, or it has been read to me. I have had the opportunity to ask questions about it and they have been answered to my satisfaction. I consent voluntarily to be a participant in this study and understand that I have the right to withdraw from the (interview/discussion) at any time. **.**

**Respondents Signature: _____________   Date: _______________________**

I, the undersigned, have fully explained the relevant details of this survey to the respondent to consent.

Enumerator’s signature: ____________________

**Date:____________________**

Enumerator’s name:**_______________________________**

**Appendix 2: CHVs Focus Group Discussion Interview Guide**

**Study title: APPLICATION OF THE ULTRA-POVERTY GRADUATION MODEL IN UNDERSTANDING COMMUNITY HEALTH VOLUNTEERS’ PREFERENCES FOR SOCIO-ECONOMIC EMPOWERMENT STRATEGIES: A QUALITATIVE STUDY IN KILIFI, KENYA**

**Welcome and Introductions by the Facilitator**

| **Task** | **Suggested Narrative** |
| --- | --- |
| Introduce self and reasons for bringing everyone together for the FGD | Hi, my name is XXXX and I am here on behalf of the Access to Quality Care through Extending and Strengthening Health Systems (AQCESS) project funded by Global Affairs Canada (GAC) and co-funded by AKF and implemented in Kaloleni by Aga Khan University (East Africa to hear your views with respect to Exploring strategies for retention of Community Health Volunteers in Kilifi County. |
| Thank everyone for attending | Thank you for coming – we are grateful for your time. We are really excited to be here with you and talk to you about your work and what the project can do support you to ensure that this important and essential work that you do as CHWs continues long after the project |
| Provide critical information about how the session will go | We would like to record these discussions to help us remember them and so that we do not miss any of the ideas you give us. The details of these discussions will not be shared with anyone else; your names will be kept confidential and no one else will know who said what during our conversation. So please feel free to express your opinions openly. |
| Confirm participation including signing of consent forms to participate | If you are not comfortable with this arrangement you do not have to participate. Would you still like to participate in this discussion? If yes, I am passing around a consent form for each of you to sign confirming that you are participating voluntarily and that you are ok with us recording the discussions |
| Confirm again importance of everyone’s engagement | Great! I first want to say that we invited all of you here, and so we would like to hear from everyone. Everyone’s ideas, experiences and opinions are important. So please, everyone please speak up! |

**Guidance questions for discussions**

Ask each of the participants to introduce themselves and share information about themselves, their age, their marital status, whether they have children, how long they have been a community health volunteer and what it is that keeps them doing the work they do.

1. Ask how community health volunteers earn income to sustain themselves in their area? What challenges do you think you are likely to face that will affect this? (Ask specifics for outcomes different from previously used or mentions IGAs)
2. Ask how their work as CHVs affects their livelihood activities? To what extent? (Please expand)
3. Ask if they have been involved in livelihood activities as a result of being CHVs, if yes: who initiated? What type of livelihood activities? Was at individual or group level? What was the outcome? What is the current status of the activity?
4. Ask them, if any opportunity existed for them to receive support to supplement their incomes, what sort of income generating activities would they like to be supported to do and why?
5. Ask them besides monetary handouts how else would you like to receive this support to enable you to undertake income generating activities? Would you like to receive this support as individual CHVs? OR as groups? Probe for reasons why?
6. Ask them whom they think would be best suited in their communities to support them to develop Income generation activities? Probe for reasons why?
7. Ask them how they think they would balance the tasks of continuing the community health work and also implementing income generation activities?
8. Ask them the kind of IGAs they prefer and probe Why these ones?
9. Apart from income generating activities, what other support would motivate them to continue working as CHVs (E.g. some may prefer training in a particular skill etc., rather than direct income earning activity).

**Appendix 3: CHVS Informant Interview guide**

**Welcome and Introductions by the Facilitator**

KEY INFORMANT INTERVIEW GUIDE

Community Themes & Strengths Assessment

Kilifi County

Interviewer’s Initials: ……………………….

Date: ………………. Start time: ……………………. End time: …………………………….

Name: …………………………………………… Title: ……………………………………..

Agency/Organization: …………………………………………………………………………

No. of years living in Kilifi County: ________ No. of years in current position: _______

Introduction: Good morning/afternoon. My name is……………………….. Thank you for taking time out of your busy day to speak with me. This should take about 1 hour of your time, but we may find that we may run over – up to 90 minutes total - once we get into the interview. (Check to see if this is okay)

I am here on behalf of the Access to Quality Care through Extending and Strengthening Health Systems (AQCESS) project funded by Global Affairs Canada (GAC) and implemented by The Aga Khan University (East Africa). I would like to hear your views with respect to Exploring strategies for retention of Community Health Volunteers in Kilifi County. In particular, we are interested in identifying viable income generating activities (IGAs) for CHVs that can enhance their retention in community health work even after the end of this project. Community input is essential to this process. Focus groups discussions and key informant interviews are being used to engage community health volunteers and other stakeholders.

You have been selected for a key informant interview because of your knowledge, insight and familiarity with the community, CHVs and the community health work in this area. The ideas that emerge from these interviews will be feed-back to all stakeholders. I would like to record these discussions to help us remember them and so that we do not miss any of the ideas, you give us. Individual interviews will be kept strictly confidential.

If you are not comfortable with this arrangement, you do not have to participate. At this juncture ask: “Would you still like to participate in this discussion?” If yes, here is a consent form for you to sign confirming that you are participating voluntarily and that you are ok with me recording the discussions. Thank the informant.

***To get us started, can you tell me briefly about the work that you and your organization do in the community?***

Should also ask about their work with/in community health and CHVs, how long, in what capacity, challenges they face/faced by CHVs in their work.

Thank you. Now I would like to ask you a series of questions about your views with regard to the retention of CHVs in Kilifi County (also Kaloleni and Rabai sub-county depending on the scope of responsibility of the interviewee). As you consider your response to these questions, I wish to request you to keep in mind the broad definition of the Kenya Community Health Strategy and the combined role of you and other stakeholders in achieving the strategic objectives namely:

1. Strengthen the delivery of integrated, comprehensive, and quality community health services for all cohorts
2. Strengthen community structures and systems for effective implementation of community health actions and services at all levels
3. Strengthen data demand and information use at all levels
4. Strengthen mechanisms for resource mobilization and management for sustainable implementation of community health services

**Guidance questions for interviews**

1. *In your role as (select: MOH county official/MoA county official/Community Health Committee Chairperson/NGO/CBO official) how often do you engage with community health volunteers? What is your specific role in this regard?*
2. *Do you in your opinion feel that there any challenges in retaining CHWs over the past years?*
3. *Why do you think (based on answer from previous question) there has been an improvement, decline, or stagnation of the same)? (Probe). Please describe these factors in detail.? A lot has been said about the fact that the work CHWs engage in is voluntary. Do you think CHVs should earn an income? Why or why not?*
4. *There has been talk about CHWs developing Income Generating Activities (IGAs) within their line of work. Do you feel this is feasible? Why or why not? (Probe) How would this affect their output?*
5. *What policies are in place/are you aware of at (select: Ministry/Organization/Community/Institution) meant to enhance sustainability or retention of CHVs in Community health work?*
6. *Are there any policies at your (select: Ministry/Organization/Community/Institution) to support CHVs supplement their incomes? How do these align with the Community Health Strategy? (Probe) [if there are no policies or they do not know of any I think questions 8, 9 and 10 may not be applicable, proceed to question 11]*
7. *In what ways, are the measures stated above to support CHVs engaging in IGAs, sustainable?*
8. *Whom specifically within the ministry/institution would the CHVs need to engage to acquire this support? How would they support them and why? How is this documented?*
9. *With regard to the proposed support to CHVs in undertaking these income generation activities, would the support be available as individual CHWs? Alternatively, as a group? (Probe for reasons why.)*
10. *Are you aware of any IGAs that have been implemented for CHVs in this area (or any other area where you have worked)? If yes, who implemented these IGAs? How were they implemented? In your opinion, would you say they were successful overall, and specifically in improving retention of CHVs?*
11. *In your experience what challenges have, you faced in supporting the continued work done by your community health volunteers. In addition, in supporting the implementation of income generation activities?*
12. *What in your opinion needs to be done to address these challenges (no.12 above)?*
13. *Are there any income generating activities you think are viable that the CHVs could be engaged in that would assist your Ministry/Institution enhance their retention in community health work? Which activities? Why do you think they are viable in this area? What would be the most feasible approach of implementing them (Probe: individual or groups, others)?*

**Closing:** Thanks so much for sharing your concerns and perspectives on these issues. The information you have provided will contribute to develop a better understanding about factors impacting health and quality of life in Kilifi County.

***Before we conclude the interview, is there anything you would like to add?***

As a reminder, summary results will be made available to you and used to inform the development of IGAs for CHVs in this area.

Should you have any questions, please feel free to contact the PI of this study, Dr Njeri Nyanja. Here is her contact information.

Email: [njeri.nyanja@aku.edu](mailto:njeri.nyanja@aku.edu)

Mobile: +254-711-092895

Thank you again for your time. It was a pleasure.

**Appendix 4: Consolidated criteria for reporting qualitative studies (COREQ): 32-item checklist**

| **No. Item** | **Guide questions/description** | **Reported on Page #** |
| --- | --- | --- |
| **Domain 1: Research team and reﬂexivity** |  |  |
| *Personal Characteristics* |  |  |
| 1. Inter viewer/facilitator | Which author/s conducted the inter view or focus group? | Methods  (SC) |
| 2. Credentials | What were the researcher’s credentials? E.g. PhD, MD | Master’s Degree |
| 3. Occupation | What was their occupation at the time of the study? | Qualitative consultant |
| 4. Gender | Was the researcher male or female? | Female |
| 5. Experience and training | What experience or training did the researcher have? | Methods |
| *Relationship with participants* |  |  |
| 6. Relationship established | Was a relationship established prior to study commencement? | N/A |
| 7. Participant knowledge of the interviewer | What did the participants know about the researcher? e.g. personal goals, reasons for doing the research | N/A |
| 8. Interviewer characteristics | What characteristics were reported about the inter viewer/facilitator? e.g. Bias, assumptions, reasons and interests in the research topic | N/A |
| **Domain 2: study design** |  |  |
| *Theoretical framework* |  |  |
| 9. Methodological orientation and Theory | What methodological orientation was stated to underpin the study? e.g. grounded theory, discourse analysis, ethnography, phenomenology, content analysis | Methods |
| *Participant selection* |  |  |
| 10. Sampling | How were participants selected? e.g. purposive, convenience, consecutive, snowball | Methods (purposive) |
| 11. Method of approach | How were participants approached? e.g. face-to-face, telephone, mail, email | Methods (face to face) |
| 12. Sample size | How many participants were in the study? | Methods (78) |
| 13. Non-participation | How many people refused to participate or dropped out? Reasons? | None |
| *Setting* |  |  |
| 14. Setting of data collection | Where was the data collected? e.g. home, clinic, workplace | Methods  (Kilifi county, Kenya) |
| 15. Presence of non-participants | Was anyone else present besides the participants and researchers? | Methods  (None) |
| 16. Description of sample | What are the important characteristics of the sample? e.g. demographic data, date | Methods  (CHVs and local leaders to understand their lived experience) |
| *Data collection* |  |  |
| 17. Interview guide | Were questions, prompts, guides provided by the authors? Was it pilot tested? | Methods |
| 18. Repeat interviews | Were repeat inter views carried out? If yes, how many? | N/A |
| 19. Audio/visual recording | Did the research use audio or visual recording to collect the data? | Methods |
| 20. Field notes | Were ﬁeld notes made during and/or after the inter view or focus group? | Methods |
| 21. Duration | What was the duration of the interviews or focus group? | Methods  (no more than one hour) |
| 22. Data saturation | Was data saturation discussed? | Methods |
| 23. Transcripts returned | Were transcripts returned to participants for comment and/or correction? | N/A |
| **Domain 3: analysis and ﬁndings** |  |  |
| *Data analysis* |  |  |
| 24. Number of data coders | How many data coders coded the data? | Methods |
| 25. Description of the coding tree | Did authors provide a description of the coding tree? | N/A |
| 26. Derivation of themes | Were themes identiﬁed in advance or derived from the data? | Methods |
| 27. Software | What software, if applicable, was used to manage the data? | NVivo |
| 28. Participant checking | Did participants provide feedback on the ﬁndings? | Strengths and limitations |
| *Reporting* |  |  |
| 29. Quotations presented | Were participant quotations presented to illustrate the themes/ﬁndings? Was each quotation identiﬁed? e.g. participant number | Results |
| 30. Data and ﬁndings consistent | Was there consistency between the data presented and the ﬁndings? | Findings and discussions |
| 31. Clarity of major themes | Were major themes clearly presented in the ﬁndings? | Results |
| 32. Clarity of minor themes | Is there a description of diverse cases or discussion of minor themes? | Results |
